# Affective Processing in Depression Risk: Insights From Event‐Related Potentials

**DOI:** 10.1111/psyp.70367

**Published:** 2026-07-27

**Authors:** Carola Dell'Acqua, Claudio Imperatori, Valentina Mologni, Rita Bianca Ardito, Letizia Soliman, Mauro Adenzato, Giuseppe Alessio Carbone, Aurelia Lo Presti, Benedetto Farina, Simone Messerotti Benvenuti

**Affiliations:** ^1^ Department of General Psychology University of Padua Padua Italy; ^2^ Experimental and Applied Psychology Laboratory, Department of Health and Life Sciences European University of Rome Rome Italy; ^3^ Padova Neuroscience Center (PNC) University of Padua Padua Italy; ^4^ Department of Psychology University of Turin Turin Italy; ^5^ Faculty of Psychology and Educational Sciences University of Geneva Geneva Switzerland

**Keywords:** depression vulnerability, emotional processing, ERPs, family history of depression, subclinical depression

## Abstract

A mechanism that might be implicated in the etiopathogenesis of depression is a blunted activation of the Positive and Negative Valence Systems, leading to a reduced elaboration of emotional stimuli. Yet, relatively little attention has been devoted to disentangling the distinct temporal phases that constitute this process, namely, emotional anticipation and emotional stimulus processing. Hence, the primary aim of the present study was to investigate distinct phases of emotional responses as potential psychophysiological correlates of vulnerability to depression. Data was collected from young adults with a high vulnerability to depression, which was determined by the presence of a family history of the condition but no current symptoms (*n* = 23, 19 F), or the presence of subclinical depressive symptoms (*n* = 39, 25 F), and 30 (19 F) controls with neither a family history nor current symptoms. Participants performed an S1–S2 task, in which an emotional picture (pleasant, neutral, unpleasant) (S2) was anticipated by a cue (S1) that predicted its valence. During the task, the electroencephalogram was recorded. Emotional anticipation was indexed by the Cue‐P300, whereas emotional elaboration was indexed by the late positive potential (LPP) at three processing windows (300–600, 600–1000, 1000–2000 ms). Both vulnerability groups showed reduced Cue‐P300 for pleasant and unpleasant cues relative to controls, indicating diminished preparatory allocation of attentional resources to upcoming emotional events. During image processing, both at‐risk groups exhibited reduced initial motivated attention (i.e., LPP amplitude in 300–600 ms time window) to pleasant, but not unpleasant, stimuli compared to controls, whereas no between‐group differences emerged in later LPP time windows. These findings suggest that depression vulnerability is characterized by broadly blunted emotional anticipation and a selective reduction in motivated attention to pleasant stimuli.

## Abstract 2

Using event‐related potentials (ERPs) in an emotional S1–S2 paradigm, this study demonstrates that both familial and subclinical vulnerability to depression are characterized by blunted anticipation of emotional stimuli and reduced motivated attention to pleasant stimuli. These effects emerged even in the absence of changes in subjective ratings, highlighting ERPs as sensitive markers of latent depression risk and potential targets for early prevention.

## 1 Introduction

Major depressive disorder (MDD) is a highly prevalent mood disorder characterized by pervasive disturbances in affective, cognitive, and physiological functioning, resulting in substantial functional impairment (American Psychiatric Association 2022). With more than 322 million individuals affected worldwide, MDD represents one of the leading contributors to the global burden of disease (Johnston et al. 2019; Liu et al. 2020). Beyond its high prevalence, the disorder is associated with reduced life expectancy (Laursen et al. 2016) and a wide range of adverse social, occupational, and health‐related outcomes (Johnson et al. 2018). When inadequately treated, MDD often follows a chronic and recurrent trajectory, with cumulative disability increasing across the lifespan. Hence, prevention of this mental disorder represents a critical public health priority. Effective preventive strategies require the early identification of individuals at heightened risk, ideally prior to the onset of a full‐blown disorder. This underscores the importance of investigating psychological and psychophysiological vulnerability markers in populations known to be at increased risk, such as individuals with a family history of depression (Gotlib et al. 2014, 2020; Gottesman and Gould 2003) or those exhibiting subclinical depressive symptoms (Lee et al. 2019; Rodríguez et al. 2012). However, progress in prevention remains limited by an incomplete understanding of the pathophysiological mechanisms underlying MDD and the processes through which risk is conferred.

One domain that has received sustained attention in research on vulnerability to depression is emotional processing, particularly the modulation of responses to pleasant and unpleasant stimuli. Within contemporary dimensional frameworks, such as the Research Domain Criteria (RDoC, Cuthbert and Insel 2013; Dell'Acqua et al. 2023), depression risk has been linked to changes in both the Positive Valence Systems (PVS) and Negative Valence Systems (NVS), which are thought to support adaptive engagement with appetitive and aversive environmental cues. Particularly, a robust body of evidence indicates that depression is characterized by blunted processing of pleasant stimuli, a finding consistently observed in individuals with clinical depression (Dell'Acqua et al. 2022; Klawohn et al. 2021; MacNamara et al. 2016; Weinberg 2023). Importantly, similar changes have also been documented in populations at elevated risk for depression, including individuals with a family history of the disorder and those with subclinical depressive symptoms (Grunewald et al. 2019; Moretta et al. 2021; Moretta and Messerotti Benvenuti 2023). These findings suggest that reduced elaboration of pleasant stimuli may represent a vulnerability marker rather than merely a consequence of the disorder.

In contrast, the literature examining emotional responses to unpleasant stimuli in depression is less consistent. While some studies have reported attentional biases or enhanced processing of unpleasant content, interpreted as evidence for increased elaboration and motivated attention to aversive stimuli (e.g., Gotlib et al. 2004; Joormann and Quinn 2014; Joormann and Siemer 2011), a growing and more convergent line of research points toward a generalized attenuation of emotional responding. Specifically, several studies have demonstrated reduced motivated attention not only to pleasant but also to unpleasant stimuli in depressed individuals and those at risk (e.g., Foti et al. 2010; Grunewald et al. 2019; Moretta and Messerotti Benvenuti 2023). This pattern of findings aligns with the Emotional Context Insensitivity (ECI) hypothesis (Rottenberg et al. 2005), which posits that depression is associated with a broad reduction in affective responsiveness to environmental stimuli, largely independent of stimulus valence.

Despite the growing interest in emotional responding as a vulnerability factor for depression, relatively little attention has been devoted to disentangling the distinct temporal phases that constitute this process, namely, emotional anticipation and emotional stimulus processing. These phases reflect partially dissociable mechanisms that may be differentially affected in depression and its risk, thereby contributing in distinct ways to the emergence and persistence of depressive symptoms (Brudner et al. 2018). On one hand, emotional anticipation refers to a preparatory process that enables individuals to prepare for potentially salient emotional events. During anticipation, attentional resources are allocated to support an adaptive response to upcoming stimuli (Bermpohl et al. 2006; Pastor et al. 2014; Schindler et al. 2014). Alterations in anticipatory processes may therefore compromise motivational engagement and readiness to respond to emotionally relevant events (Schindler et al. 2014). On the other hand, emotional stimulus processing represents a subsequent phase characterized by an initial perceptual orienting toward the affective stimulus, followed by sustained, motivated attention that is dynamically modulated by the stimulus' motivational salience (Castelfranchi and Miceli 2010; Schindler et al. 2014). Disruptions at this stage may manifest as reduced elaboration of emotionally salient information, limiting the individual's capacity to effectively process and integrate affective content from the environment. A more fine‐grained investigation of these components has the potential to advance our understanding of the mechanisms underlying depression.

At the psychophysiological level, event‐related potentials (ERPs) offer a powerful tool for investigating emotional processing due to their excellent temporal resolution, which allows for the dissociation of temporally distinct processes. A particularly informative paradigm for investigating both anticipatory and elaboration phases of emotional processing is the S1–S2 task (e.g., Poli et al. 2007). In this paradigm, an initial cue (S1) conveys predictive information regarding the affective valence of a subsequent imperative stimulus (S2). This design enables the dissociation of multiple components of emotional processing, spanning anticipatory, and stimulus‐engagement stages. Specifically, three ERP components are of primary interest: the cue‐related P300 (Cue‐P300), the Stimulus‐Preceding Negativity (SPN), and the late positive potential (LPP). The Cue‐P300, time‐locked to the presentation of the cue (S1), reflects the allocation of attentional resources toward an upcoming emotionally salient stimulus and indexes the engagement of motivated attention in anticipation of affective events (Glazer et al. 2018; Novak et al. 2016; Novak and Foti 2015). The SPN is a slow negative‐going potential that emerges in the interval preceding stimulus onset and is commonly observed approximately 200 ms before the presentation of the imperative stimulus (S2). This component is thought to reflect affective expectancy and anticipatory processing related to emotionally salient outcomes (Brunia and Van Boxtel 2001; Poli et al. 2007). Following stimulus presentation, emotional engagement is indexed by the LPP, a sustained positive deflection occurring approximately 300–1000 ms after stimulus onset, which reflects motivated attention toward affective–motivationally salient stimuli (Hajcak and Foti 2020; Palomba et al. 1997; Schupp et al. 2000). According to Hajcak and Foti (2020), the LPP reflects output from a common neural system that responds to stimulus significance. Indeed, an increased LPP has been consistently reported in response to emotional stimuli compared to neutral stimuli (Palomba et al. 1997; Hajcak and Foti 2020; Schupp et al. 2000).

Building on this approach, there is evidence that depression might be associated with changes in anticipatory processes. Specifically, recent studies have reported reduced Cue‐P300 amplitudes during reward‐based tasks (Thompson et al. 2023), as well as associations between diminished SPN amplitudes and greater severity of depressive symptoms (Luckhardt et al. 2023; Ren et al. 2024). These findings underscore the potential role of anticipatory dysfunction in depression. However, evidence in at‐risk samples remains scarce.

With respect to the elaborative stage, attenuated LPP amplitudes to emotional stimuli have been consistently observed in individuals with depression and in populations at risk for the disorder. This pattern has been reported in individuals with clinical and subclinical depressive symptoms (Grunewald et al. 2019; Klawohn et al. 2021; Levinson et al. 2019; Moretta et al. 2021), as well as in those with a family history of depression (Moretta and Messerotti Benvenuti 2023). Reductions are reliably found for pleasant stimuli (e.g., Angeleri et al. 2026; Grunewald et al. 2019; Moretta et al. 2021) and, less consistently, for unpleasant stimuli (Foti et al. 2010; Moretta and Messerotti Benvenuti 2023). Consistent with this pattern, a recent meta‐analysis showed that adults with depressive symptoms exhibit overall reduced LPP amplitudes to emotional stimuli compared to controls (Dell'Acqua et al. 2026). However, the extent to which such alterations are already present in individuals at elevated risk (i.e., subclinical depression or familial vulnerability) remains insufficiently explored.

In light of these premises, the primary aim of the present study was to investigate distinct phases of emotional processing as potential psychophysiological correlates of vulnerability to depression. To this end, electroencephalographic (EEG) activity was recorded while participants completed an S1–S2 passive viewing task involving affective images (pleasant, neutral, and unpleasant) selected from the International Affective Picture System (IAPS; Lang et al. 1997). The sample consisted of young adults divided into two groups characterized by elevated vulnerability to depression: individuals with a family history of depression and individuals with subclinical depressive symptoms, and a control group with no current symptoms or known vulnerability to the disorder. Consistent with the existing literature and with the theoretical assumptions of the ECI model (Bylsma et al. 2008; Bylsma 2021; Rottenberg et al. 2005), the following hypotheses were formulated.

1. It was hypothesized that participants in the two vulnerability groups (familial risk and subclinical depression) would exhibit reduced anticipatory and stimulus‐related processing of emotional stimuli, regardless of valence. Specifically, this reduction was expected to be reflected in smaller amplitudes of the Cue‐P300 and SPN during the anticipatory phase, as well as reduced LPP amplitudes during the processing of pleasant and unpleasant stimuli, compared to the control group without vulnerability to depression.
2. It was further hypothesized that, in the control group, emotional images (both pleasant and unpleasant) would elicit greater anticipatory and stimulus‐related processing than neutral images, as indexed by larger Cue‐P300, SPN, and LPP amplitudes. In contrast, in participants with subclinical depression and those with a family history of depression, the amplitudes of the Cue‐P300, SPN, and LPP in response to pleasant and unpleasant stimuli were expected not to differ significantly from those elicited by neutral stimuli, indicating attenuated anticipation and processing of affective information.

## 2 Materials and Methods

### 2.1 Participants

A volunteer sample of 92 young adults (63 females; mean age = 22.85 ± 3.02 years, range = 19–35) was recruited between November 2023 and November 2025, primarily from the University of Padua student population.^1^ A prospective simulation‐based power analysis was conducted in R using the *simr* package (Green and MacLeod 2016) to estimate the sample size required to detect the Category × Group interaction in the planned linear mixed‐effects model (DV ~ Category × Group + (1|Participant)). Data were simulated under a 3 (Group: control, subclinical depression, familial risk) × 3 (Category: pleasant, neutral, unpleasant) design with random intercepts for participants. Assuming a medium‐sized interaction effect (*d* = 0.50) and variance parameters consistent with ERP research, statistical power was estimated via likelihood‐ratio tests (*α* = 0.05) across increasing sample sizes. Power was estimated using Monte Carlo simulations and likelihood‐ratio tests of the Category × Group interaction. The analysis indicated that approximately *n* = 80 participants in total are required to achieve 80% power to detect the Category × Group interaction. The present sample (*n* = 92) therefore provides adequate power under a medium effect size assumption.

All participants were screened to exclude the presence of neurological, mental, or other relevant medical conditions and were not taking psychotropic medication, as verified through an online screening questionnaire followed by an ad hoc clinical interview. Participants were excluded if they reported a family history of psychotic disorders, a current major depressive episode at the time of assessment, or the use of psychotropic substances or medications within the two weeks preceding participation.

Based on predefined criteria, the sample was divided into three groups: two groups characterized by elevated vulnerability to depression and one control group. The vulnerability groups included individuals with a family history of depression in the absence of current depressive symptoms and individuals presenting with subclinical depressive symptoms. The control group comprised participants without depressive symptoms or familial vulnerability to depression. The presence of current or past depressive episodes was assessed using Module A of the Structured Clinical Interview for DSM‐5 Disorders‐Clinician Version (SCID‐5‐CV; First et al. 2017; Italian version by Fossati and Borroni 2017). Depressive symptom severity was further evaluated using the Beck Depression Inventory–II (BDI‐II; Beck et al. 1987; Sica and Ghisi 2007). Familial risk for depression was assessed using the Family History Screen (FHS; Weissman et al. 2000), which allowed for the identification of depressive disorders in first‐degree relatives (parents, siblings, children) and the exclusion of a family history of other psychiatric conditions (see, for example, Dell'Acqua et al. 2024; Moretta and Messerotti Benvenuti 2023).

Participants were assigned to the group with subclinical depression if they reported at least two depressive symptoms on the SCID‐5‐CV Module A and obtained a BDI‐II score of 12 or higher, without meeting criteria for a major depressive episode (*n* = 39; 25 females). The group with a family history of depression included participants with at least one first‐degree relative with a history of depression, no current depressive symptoms on the SCID‐5‐CV, and a BDI‐II score below 12 (*n* = 23; 19 females). The control group consisted of participants without a family history of depression, without depressive symptoms on the SCID‐5‐CV, and with a BDI‐II score below 12 (*n* = 30; 19 females). In the final sample, a history of past depression was reported by five participants in the control group, 11 participants in the family history group, and 18 participants in the subclinical depression group.

All participants provided written informed consent prior to participation. The study was conducted in accordance with the Declaration of Helsinki and was approved by the Ethics Committee for Psychological Research, Area 17, University of Padua (protocol no. 220‐c). Participants did not receive any financial or material compensation for their participation.

### 2.2 Psychological Measures and Experimental Task

During data collection, one self‐report questionnaire (BDI‐II; Beck et al. 1996; Sica and Ghisi 2007) and two semi‐structured interviews (SCID‐5‐CV; First et al. 2017; FHS; Weissman et al. 2000) were administered.

The Italian version of the BDI‐II (Sica and Ghisi 2007) is a 21‐item self‐report questionnaire designed to assess the presence and severity of depressive symptoms in adolescent and adult populations (≥ 13 years; Beck et al. 1996). Each item evaluates a specific depressive symptom and its perceived severity over the previous two weeks using a 4‐point Likert scale (0–3). Total scores range from 0 to 63, with higher scores indicating greater symptom severity. In accordance with Italian normative data, a cut‐off score of 12 was used to identify the presence of subclinical depressive symptoms (Sica and Ghisi 2007). In the present sample, the BDI‐II showed excellent internal consistency, Cronbach's *α* = 0.91.

The Italian version of Module A for mood disorders of the SCID‐5‐CV (First et al. 2017) was administered for research purposes to assess subclinical depressive symptomatology and to exclude participants with a current major depressive or manic episode. The interview was conducted by a licensed and trained clinical psychologist.

The FHS (Weissman et al. 2000) is a standardized semi‐structured interview consisting of 17 items designed to assess the presence of current or lifetime psychiatric disorders in first‐degree relatives (parents, siblings, children). The interview is administered directly to the participant, who provides information regarding their family members. Disorders assessed include substance use disorders, major depressive disorder, anxiety disorders, bipolar disorder, psychotic disorders, and suicidal behaviors (Weissman et al. 2000). Familial risk for depression was operationalized based on participants' responses to the FHS. Specifically, a positive family history of depression was defined by the presence of at least one first‐degree relative for whom the participant endorsed either Item 7 (“Has any of the relatives ever felt sad or depressed for most of the day for at least two weeks?”) or Item 8 (“Has any of the relatives ever experienced periods of feeling tired, with less energy and reduced interest in usual activities for at least two weeks?”), in line with previous e.g., (Dell'Acqua et al. 2024; Moretta and Messerotti Benvenuti 2022, 2023; Khoubaeva et al. 2023; Watters et al. 2013).

### 2.3 Experimental Task

Participants completed an affective S1–S2 task during continuous EEG recording (see, e.g., Dell'Acqua et al. 2025). Stimuli were presented on a 16‐in. monitor positioned approximately one meter from the participant. The task consisted of 72 trials and was administered using E‐Prime software (Psychology Software Tools).

Each trial began with a 500‐ms baseline period, during which a white fixation point was displayed on a gray background. This was followed by the presentation of a cue (S1) lasting 1500 ms, which provided predictive information regarding the emotional valence of the upcoming image (S2): a minus sign (−) indicated an unpleasant image, a plus sign (+) indicated a pleasant image, and a circle indicated a neutral image. After an inter‐stimulus interval (ISI) of 4500 ms, the affective image (S2) was presented for 2000 ms. Each emotional stimulus was followed by a variable inter‐trial interval (ITI) ranging from 3500 to 4500 ms, during which a white fixation point was displayed on the screen.

During the experimental task, participants were instructed to carefully observe the symbols and images presented on the screen (S1 and S2). The task did not require any motor response, allowing for the assessment of spontaneous anticipatory and stimulus‐related neural activity without confounding response‐related processes.

The 72 color images (600 × 800 pixels) used as imperative stimuli (S2) were selected from the IAPS (Lang et al. 2005).^2^ Stimuli were categorized based on affective valence and arousal level and were divided into three experimental conditions, each comprising 24 images: pleasant and unpleasant images (both characterized by high arousal) and neutral images (characterized by low arousal). Pleasant images included explicitly erotic scenes and depictions of sports, whereas unpleasant images consisted of threatening content, such as armed assaults or attacking animals. Neutral images depicted urban landscapes, everyday objects, or individuals in emotionally neutral contexts. Pleasant and unpleasant stimuli were selected to have comparable normative arousal levels (pleasant: *M* = 6.50 ± 0.40; unpleasant: *M* = 6.50 ± 0.50; *p* = 0.98), both of which were significantly higher than those of neutral stimuli (*M* = 2.90 ± 0.70; *p* < 0.001). Pleasant images had significantly higher valence ratings than both unpleasant and neutral images (*p*s < 0.001), and neutral images had significantly higher valence scores than unpleasant images (*p* < 0.001; pleasant: *M* = 6.96 ± 0.47; neutral: *M* = 4.90 ± 0.26; unpleasant: *M* = 2.91 ± 0.65).

At the end of the experimental task, a subset of 36 images (12 per emotional category), previously presented during the task, was shown again to participants. Participants were asked to rate each image in terms of perceived valence and emotional arousal using a computerized 9‐point version of the Self‐Assessment Manikin (SAM; Bradley and Lang 1994). Due to technical problems, SAM ratings of 12 participants were not saved (2 controls, 5 with subclinical depression, 5 with familiarity for depression).

### 2.4 Procedure

Participants were recruited following the completion of an online screening form that included demographic and anamnestic questions, as well as the BDI‐II. Individuals who met the inclusion criteria were contacted via email to schedule the laboratory session and were instructed to refrain from consuming alcohol, caffeine, or recreational drugs in the hours preceding the experimental session. Upon arrival at the laboratory, participants carefully read and signed the informed consent form for study participation and data processing. They were then administered a brief anamnestic interview to verify the accuracy of the information provided in the online screening, followed by the administration of the two semi‐structured interviews (FHS and SCID‐5‐CV). Participants were subsequently seated in a sound‐attenuated and electrically shielded room, in a comfortable chair positioned in front of a computer monitor used for the experimental task. Participants were made comfortable and informed about the steps involved in electrode placement and task execution. Following the placement of electrooculogram (EOG) electrodes and the 32‐channel EEG cap, the experimenter provided detailed instructions for the S1–S2 task, which were also displayed on the computer screen. The experimental task was preceded by three practice trials (one for each emotional condition) to ensure that participants had fully understood the instructions and had become familiar with the procedure. The entire experimental session lasted approximately 90 min. At the conclusion of the task, participants completed the SAM.

At the end of the session, participants were debriefed about the purpose of the task and were asked whether they experienced any discomfort or negative emotional state following the presentation of the affective stimuli. When needed, the experimenter provided a brief supportive conversation, and participants were given time to remain in the laboratory until they felt comfortable leaving.

### 2.5 EEG Recording and Data Reduction

EEG activity was recorded using a 32‐channel elastic cap (Waveguard, ANT Neuro, Enschede, The Netherlands) equipped with 8‐mm silver/silver chloride (Ag/AgCl) electrodes. Electrodes were positioned according to the International 10–20 System (Jasper 1958) at the following sites: Fp1, Fpz, Fp2, F7, F3, Fz, F4, F8, FC5, FC1, FC2, FC6, C3, Cz, C4, CP5, CP1, CPz, CP2, CP6, T7, T8, P7, P3, Pz, P4, P8, POz, O1, Oz, and O2. EEG signals were recorded using a monopolar montage with CPz as the online reference. Electrode impedances were kept below 10 kΩ throughout the recording. Vertical EOG activity was recorded in a bipolar configuration using two Ag/AgCl electrodes placed above and below the right eye, allowing for the identification of ocular artifacts related to vertical eye movements. EEG and EOG signals were acquired in direct current mode with a sampling rate of 1000 Hz and a low‐pass hardware filter set at 40 Hz.

EEG data were subsequently downsampled to 500 Hz and preprocessed using the Brainstorm and EEGLAB toolboxes (Delorme and Makeig 2004; Tadel et al. 2011) implemented in MATLAB. A band‐pass filter between 0.01 and 30 Hz was applied, and data were re‐referenced offline to the average of the bilateral mastoids (M1 and M2). Ocular artifacts were corrected using Independent Component Analysis (ICA). For the Cue‐P300 and SPN, EEG data were segmented into 8300‐ms epochs ranging from 300 ms before S1 onset to the end of the trial. For the LPP, EEG data were segmented into 2800‐ms epochs ranging from 300 ms before to 2500 ms after S2 onset. A semi‐automatic procedure was used to identify and reject artifact‐contaminated epochs, using a voltage difference threshold of ±100 μV. Remaining epochs were visually inspected to exclude residual artifacts. Baseline correction was applied using the mean activity in the −250 to −50 ms interval preceding stimulus onset (S1 for Cue‐P300 and SPN; S2 for the LPP). Remaining epochs were subsequently inspected visually to ensure the exclusion of residual artifacts. The three groups did not differ in the percentage of rejected cue‐ and image‐locked epochs (all *p*s > 0.32; Table S1 in the Supporting Information shows the percentage of rejected epochs for cue‐ and image‐locked epochs). Visual inspection of the grand averages confirmed a predominance of Cue‐P300 and the LPP activity over parietal sites, consistent with previous studies (Klawohn et al. 2021; Novak and Foti 2015). Accordingly, a parietal electrode cluster (P3, Pz, P4) was used for both components. For the Cue‐P300, mean amplitude was extracted within the 200–400 ms window following S1 onset. For the LPP, mean amplitude was extracted within three time windows: 300–600, 600–1000, and 1000–2000 ms following S2 onset. In agreement with previous research (e.g., Buodo et al. 2012; Poli et al. 2007), the SPN was scored as the mean amplitude in the 200 ms preceding the image onset at electrodes at frontal sites. However, contrary to previous findings (e.g., Ren et al. 2024), the SPN component was not observed in the present study and was therefore not included in the statistical analyses. Reliability indices of the ERPs are reported in the Supporting Information (Table S2).

### 2.6 Statistical Analyses

All statistical analyses were conducted using RStudio (R Core Team 2023). The threshold for statistical significance was set at *p* < 0.05.

To examine the effects of Category (pleasant, neutral, unpleasant), Group (controls, subclinical depression, familial risk for depression), and their interaction (Category × Group) on subjective ratings of valence and arousal and ERPs amplitude (Cue‐P300, LPP), linear mixed‐effects models (LMMs, Bates et al. 2014) were fitted using the *lme4* package (Bates et al. 2014). In all models, the dependent variable was either the subjective SAM rating (valence or arousal) or the ERP (Cue‐P300 or LPP). Category, Group, and their interaction were entered as fixed effects. A random intercept for participant was included to account for within‐subject variability. The models were specified as follows:

$$\begin{array}{c} \text{Model}\leftarrow \text{lmer}\;(\mathrm{SAM}\;\left[\text{valence or arousal}\right]\;\text{or}\;\mathrm{ERP}\;\\ \left[\mathrm{Cue}-P300\;\text{or}\;\mathrm{LPP}\right]\sim \text{Category}\times \text{Group}+\left(1\;|\;\text{Participant}\right)) \end{array}$$

For each fixed effect, degrees of freedom, *F* values, *p* values, and confidence intervals were obtained using the *Anova* function from the *lme4* package (Bates et al. 2014). Significant effects were further explored using Tukey‐adjusted post hoc comparisons implemented via the *emmeans* package (Lenth 2023).

## 3 Results

### 3.1 Sample Characteristics

The two vulnerability groups and the control group did not differ significantly in age (control group, mean (*M*) = 22.4 ± 2.27; subclinical depression group, *M* = 22.7 ± 3.42; family history group, *M* = 23.7 ± 3.17, *p* = 0.28) or sex (*p* = 0.24). As expected, based on the inclusion criteria, BDI‐II scores differed significantly across groups (*p* < 0.001; control group, mean (*M*) = 5.2 ± 2.77; subclinical depression group, *M* = 20.4 ± 6.91; family history group, *M* = 6.04 ± 3.77). Post hoc analyses indicated that the subclinical depression group reported significantly higher BDI‐II scores than both the familial risk group and the control group (all *p*s < 0.001), whereas no significant differences emerged between the latter two groups (*p* = 0.82). Of note, among the group with subclinical depression, 18 (46%) participants reported having a family history for depression.

Table 1 shows the mean and standard deviations for each ERPs in the three groups. Table S3 shows zero‐order correlations between ERPs and age, sex, and BDI‐II scores.

**TABLE 1:** Means and standard deviation of the Cue‐P300 and LPP for each category in the three groups.

	Controls (*n* = 30)			Subclinical depression (*n* = 39)			Family history of depression (*n* = 23)		
	Pleasant	Neutral	Unpleasant	Pleasant	Neutral	Unpleasant	Pleasant	Neutral	Unpleasant
Cue‐P300	7.77 (3.5)	4.71 (2.65)	6.55 (2.79)	5.28 (3.15)	3.68 (3.01)	4.33 (3.00)	5.25 (2.76)	3.17 (1.98)	4.48 (2.24)
LPP (300–600 ms)	13.00 (5.35)	5.76 (4.56)	10.30 (5.23)	10.40 (5.85)	4.08 (5.01)	9.39 (5.96)	10.00 (5.53)	3.62 (3.55)	9.57 (5.09)
LPP (600–1000 ms)	11.20 (5.67)	4.82 (3.40)	10.70 (5.77)	10.10 (5.24)	4.73 (4.49)	11.40 (6.31)	9.95 (4.01)	3.47 (2.89)	11.40 (4.65)
LPP (1000–2000 ms)	6.86 (4.27)	2.30 (3.70)	7.00 (4.50)	5.92 (5.06)	2.40 (4.57)	7.51 (5.11)	7.05 (5.09)	2.87 (3.42)	8.75 (4.45)

### 3.2 Valence and Arousal Ratings

Table 2 shows the means and standard deviation of valence and arousal ratings for each category in the three groups.

**TABLE 2:** Mean and standard deviation of the SAM arousal and valence ratings in each group.

	Controls (*n* = 30)			Subclinical depression (*n* = 39)			Family history of depression (*n* = 23)		
	Pleasant	Neutral	Unpleasant	Pleasant	Neutral	Unpleasant	Pleasant	Neutral	Unpleasant
Valence	6.71 (0.19)	5.10 (0.12)	2.76 (0.21)	6.59 (0.18)	5.24 (0.16)	3.30 (0.22)	6.63 (0.22)	5.21 (0.15)	2.46 (0.17)
Arousal	5.08 (0.32)	2.19 (0.17)	5.91 (0.37)	4.73 (0.25)	2.17 (0.22)	5.10 (0.31)	5.38 (0.35)	2.29 (0.30)	6.04 (0.37)

*Note:* Values are expressed in mean (standard deviation).

Analyses of subjective valence ratings revealed a significant main effect of Category. No significant main effect of Group and no significant Category × Group interaction was observed (Table 3). Post hoc comparisons for the Category factor indicated that pleasant images were rated as significantly more positive than both neutral and unpleasant images (all *p*s < 0.001). Additionally, unpleasant images were rated as significantly less positive than neutral images (*p* < 0.001).

**TABLE 3:** ANOVA summary of the two linear mixed‐effects models predicting SAM arousal and valence ratings.

	df	*F*	*p*
Valence model			
Category	**2**	**305.55**	**< 0.001**
Group	2	0.88	0.41
Group × Category	4	1.05	0.38
Arousal model			
Category	**2**	**253.39**	**< 0.001**
Group	2	1.32	0.27
Group × Category	4	1.66	0.16

*Note:* Significant effects are shown in bold.

Abbreviation: df, degrees of freedom.

Analyses of subjective arousal ratings revealed a significant main effect of Category, whereas no significant main effect of Group and no significant Group × Category interaction were observed (Table 3). Post hoc comparisons for the Category factor indicated that, across all three groups, both pleasant and unpleasant stimuli were perceived as significantly more arousing than neutral stimuli (all *p*s < 0.001). In addition, pleasant stimuli were rated as significantly less arousing than unpleasant stimuli (*p* < 0.001).

### 3.3 Emotional Anticipation (Cue‐P300)

**FIGURE 1:**
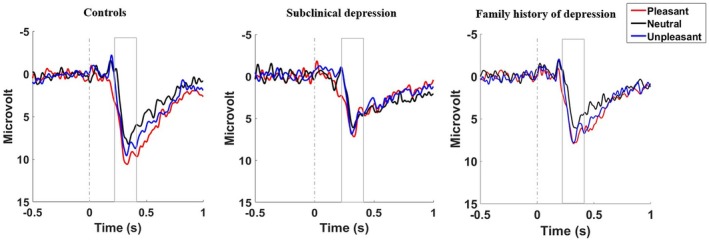
Grand average ERP waveforms during the S1–S2 task presented for the average of parietal electrodes (P3, PZ, P4). Cue (S1) onset was at 0 s (dotted line). The Cue‐P300 was scored as the mean amplitude in the gray window (200–400 ms).

**FIGURE 2:**
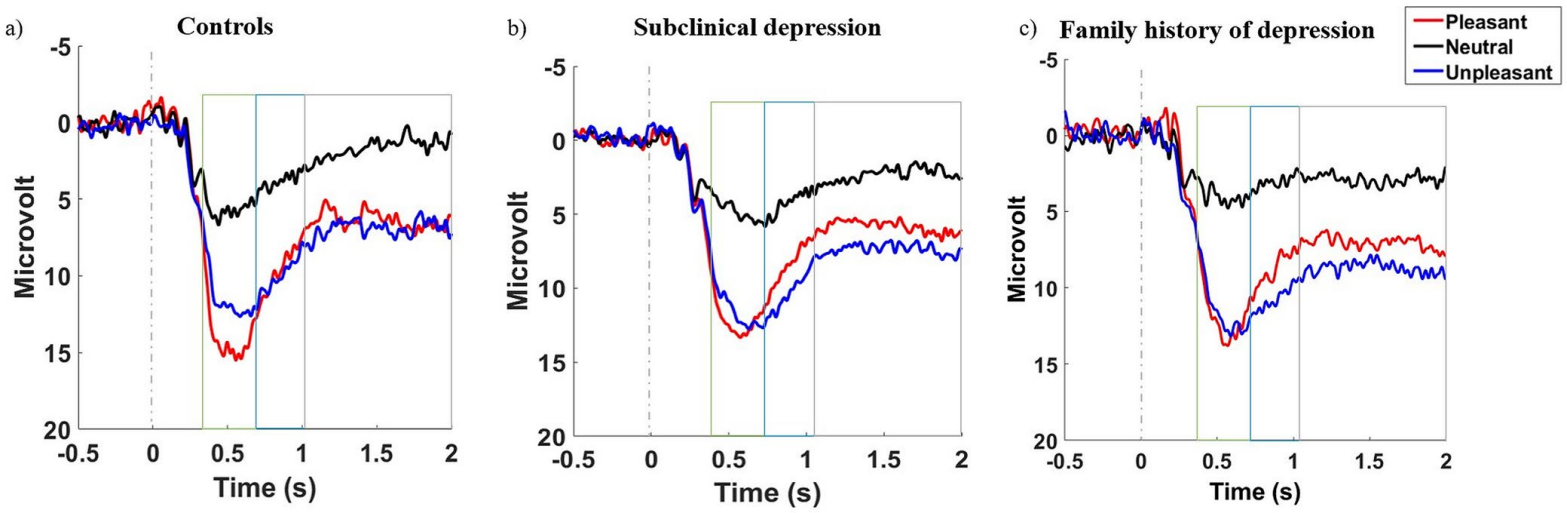
Grand average ERP waveforms during the S1–S2 task presented for the average of parietal electrodes (P3, PZ, P4). Image (S2) onset was at 0 s (dotted line). The LPP was scored as the mean amplitude in three time windows: 300–600 ms (green window), 600–1000 ms (blue window), and 1000–2000 ms (gray window).

**FIGURE 3:**
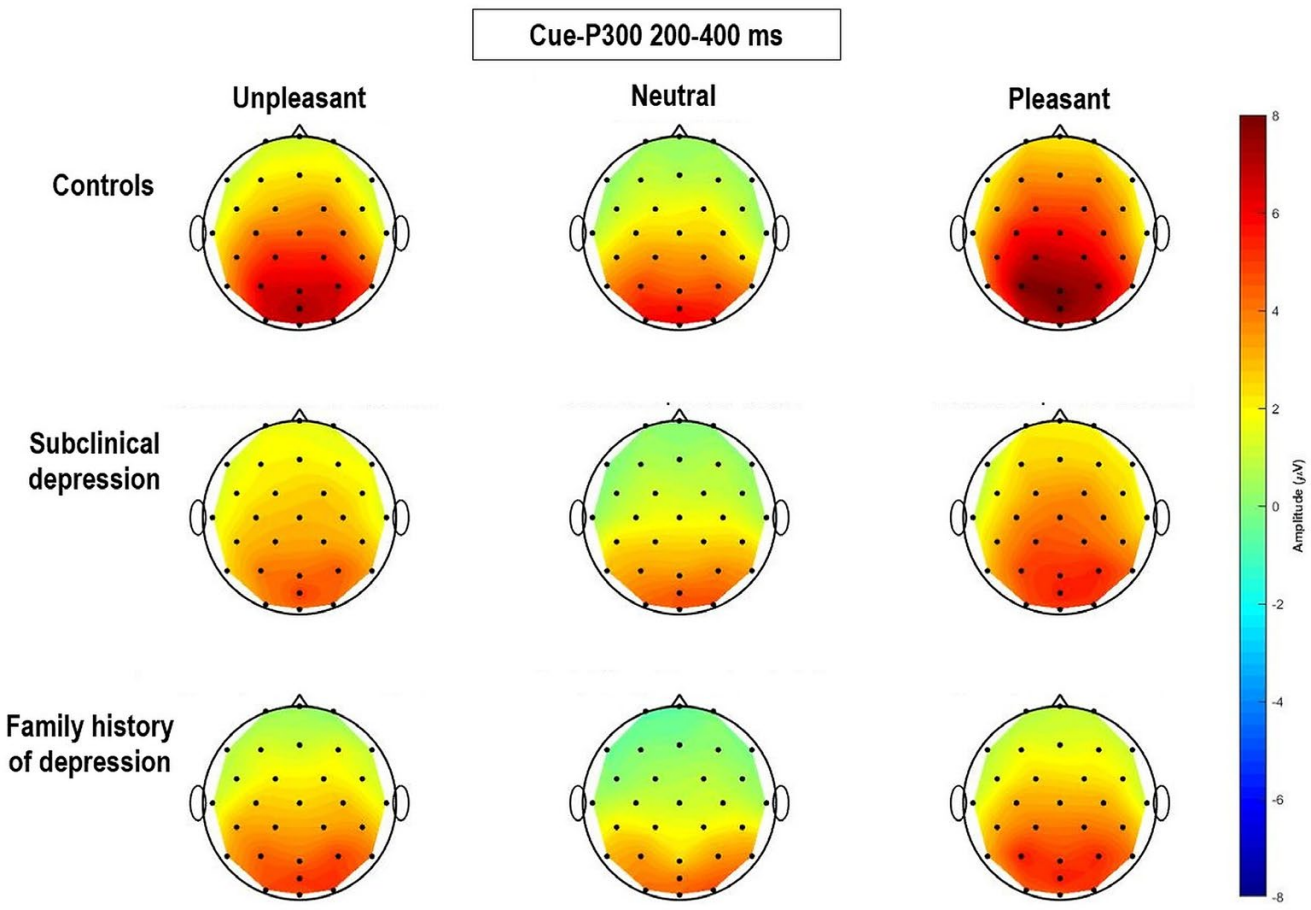
Scalp topographies of Cue‐P300 mean amplitudes in the 200–400 ms time window following cue, S1, onset during the S1–S2 task. Topographic maps are displayed separately for each group: controls, subclinical depression, and family history of depression, and for each cue category: unpleasant, neutral, and pleasant. Warmer colors indicate larger positive amplitudes in μV.

**FIGURE 4:**
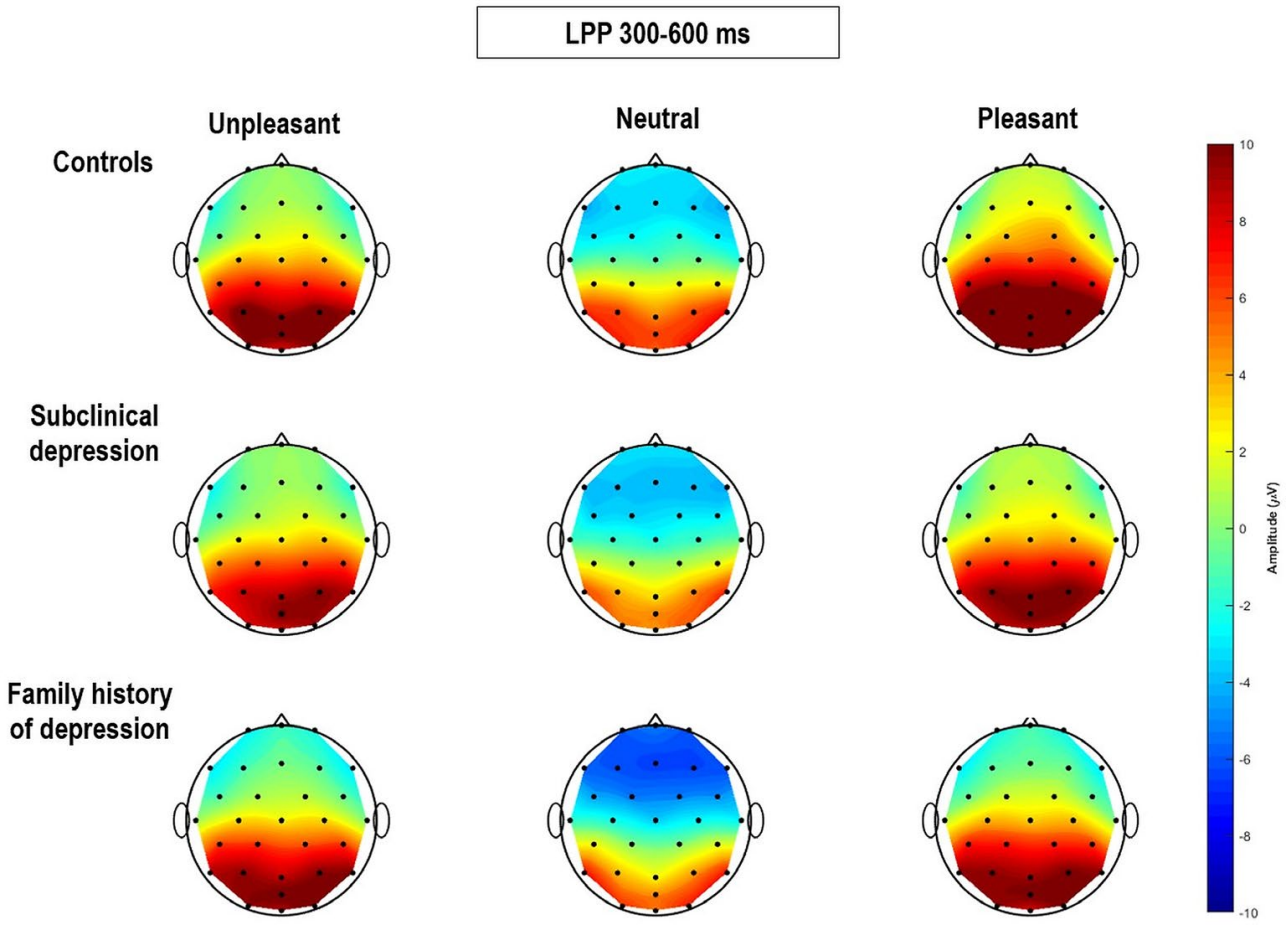
Scalp topographies of LPP mean amplitudes in the 300–600 ms time window following image, S2, onset during the S1–S2 task. Topographic maps are displayed separately for each group: controls, subclinical depression, and family history of depression, and for each image category: unpleasant, neutral, and pleasant. Warmer colors indicate larger positive amplitudes in μV.

Figures 1 and 2 show the grand average waveforms of the Cue‐P300 and LPP, respectively, for each category, unpleasant, neutral, and pleasant, in each group: controls, subclinical depression, and familial risk for depression. Figures 3 and 4 show the corresponding scalp topographies for the Cue‐P300 and early LPP (300–600 ms), respectively, across the same categories and groups. Scalp topographies for the later LPP time windows are shown in the Supporting Information (Figures S1 and S2).

**FIGURE 5:**
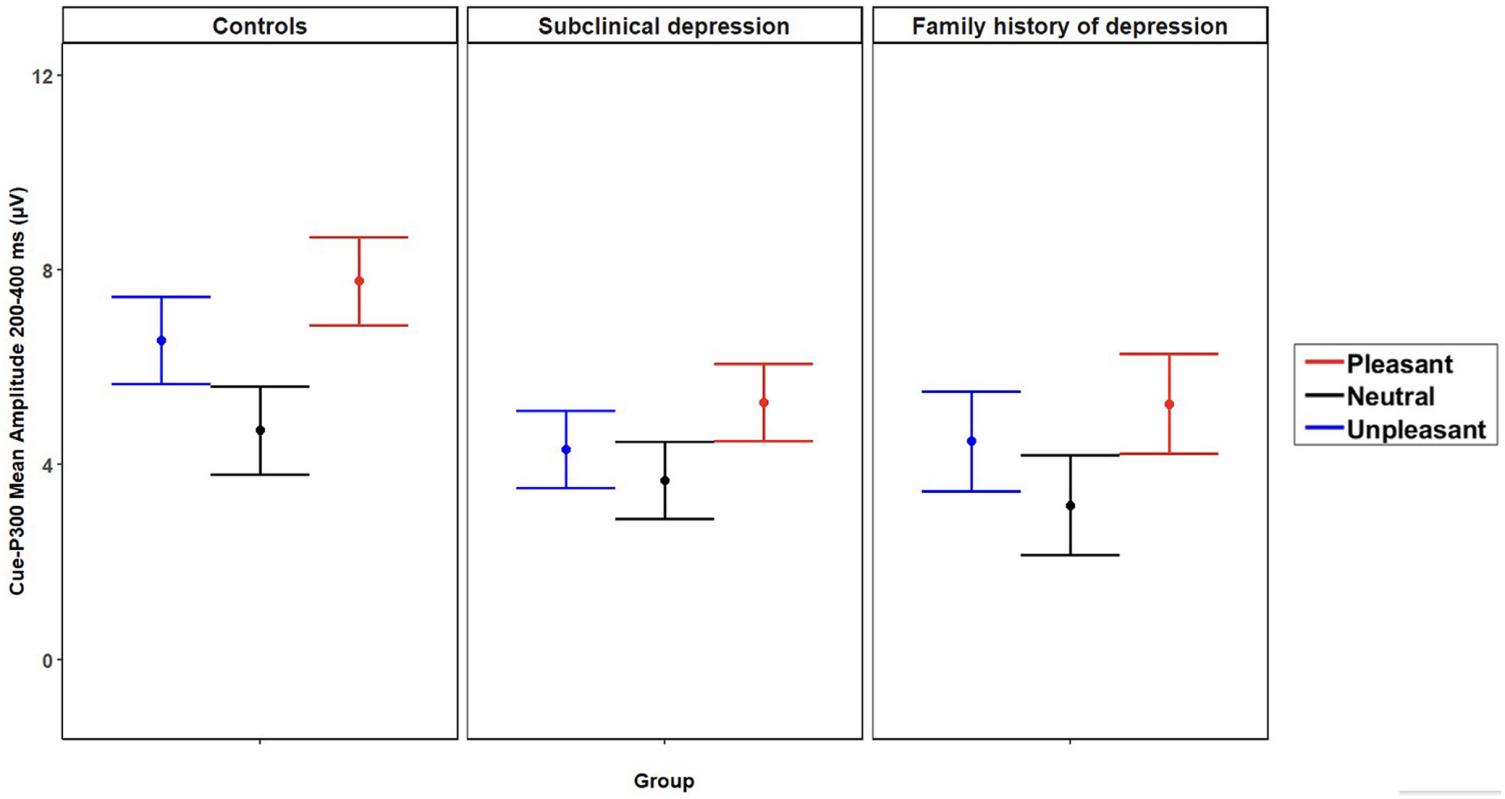
Mean amplitude of the Cue‐P300 as a function of category (pleasant, neutral, unpleasant) during cue (S1) presentation in the S1–S2 task, across the three groups: Control group (left), subclinical depression group (center), and familial risk group (right). Error bars represent the standard deviation around the mean.

Table 4 shows the results of the linear mixed‐effects model examining Cue‐P300 amplitude as a function of Category, Group, and their interaction (Group × Category). Analyses of fixed effects revealed significant main effects of Category and Group, as well as a significant Group × Category interaction (see Figure 5).

**TABLE 4:** ANOVA summary of the two linear mixed‐effects models predicting ERPs (Cue‐P300 and LPP).

	df	*F*	*p*
Cue‐P300 model			
Category	**2**	**72.27**	**< 0.001**
Group	**2**	**7.42**	**0.001**
Group × Category	**4**	**3.41**	**0.009**
LPP model (300–600 ms)			
Category	**2**	**377.06**	**< 0.001**
Group	2	1.41	0.25
Group × Category	**4**	**3.30**	**0.011**
LPP model (600–1000 ms)			
Category	**2**	**381.30**	**< 0.001**
Group	2	0.16	0.86
Group × Category	**4**	**4.18**	**0.002**
LPP model (1000–2000 ms)			
Category	**2**	**179.80**	**< 0.001**
Group	2	0.51	0.60
Group × Category	4	1.86	0.12

*Note:* Significant effects are shown in bold.

Abbreviation: df, degrees of freedom.

Post hoc within‐subjects comparisons of the significant Group × Category interaction showed that, in the control group, Cue‐P300 amplitudes were significantly larger for pleasant and unpleasant emotional trials compared to neutral trials and were larger for pleasant compared to unpleasant trials (all *p*s < 0.001). In the subclinical depression group, Cue‐P300 amplitudes were significantly larger for pleasant trials compared to both neutral and unpleasant trials (all *p*s < 0.002), whereas the difference between unpleasant and neutral trials was marginally significant (*p* = 0.057). In the familial risk group, Cue‐P300 amplitudes were significantly larger for pleasant and unpleasant trials compared to neutral trials (all *p*s < 0.001), whereas the difference between pleasant and unpleasant trials was not significant (*p* = 0.092).

Post hoc between‐subjects comparisons revealed reduced Cue‐P300 amplitudes for both pleasant and unpleasant trials in the two vulnerability groups relative to controls (all *p*s < 0.004). For neutral trials, Cue‐P300 amplitudes were comparable between controls and the subclinical depression group (*p* = 0.092), whereas the familial risk group exhibited a significantly reduced Cue‐P300 relative to controls (*p* = 0.029). No significant differences in Cue‐P300 amplitude were observed between the two vulnerability groups across any emotional category (all *p*s > 0.44).

### 3.4 Emotional Processing (LPP)

Table 4 shows the results of the linear mixed‐effects model examining LPP mean amplitude as a function of Category, Group, and their interaction (Group × Category) in all three time windows (300–600, 600–1000, 1000–2000).

**FIGURE 6:**
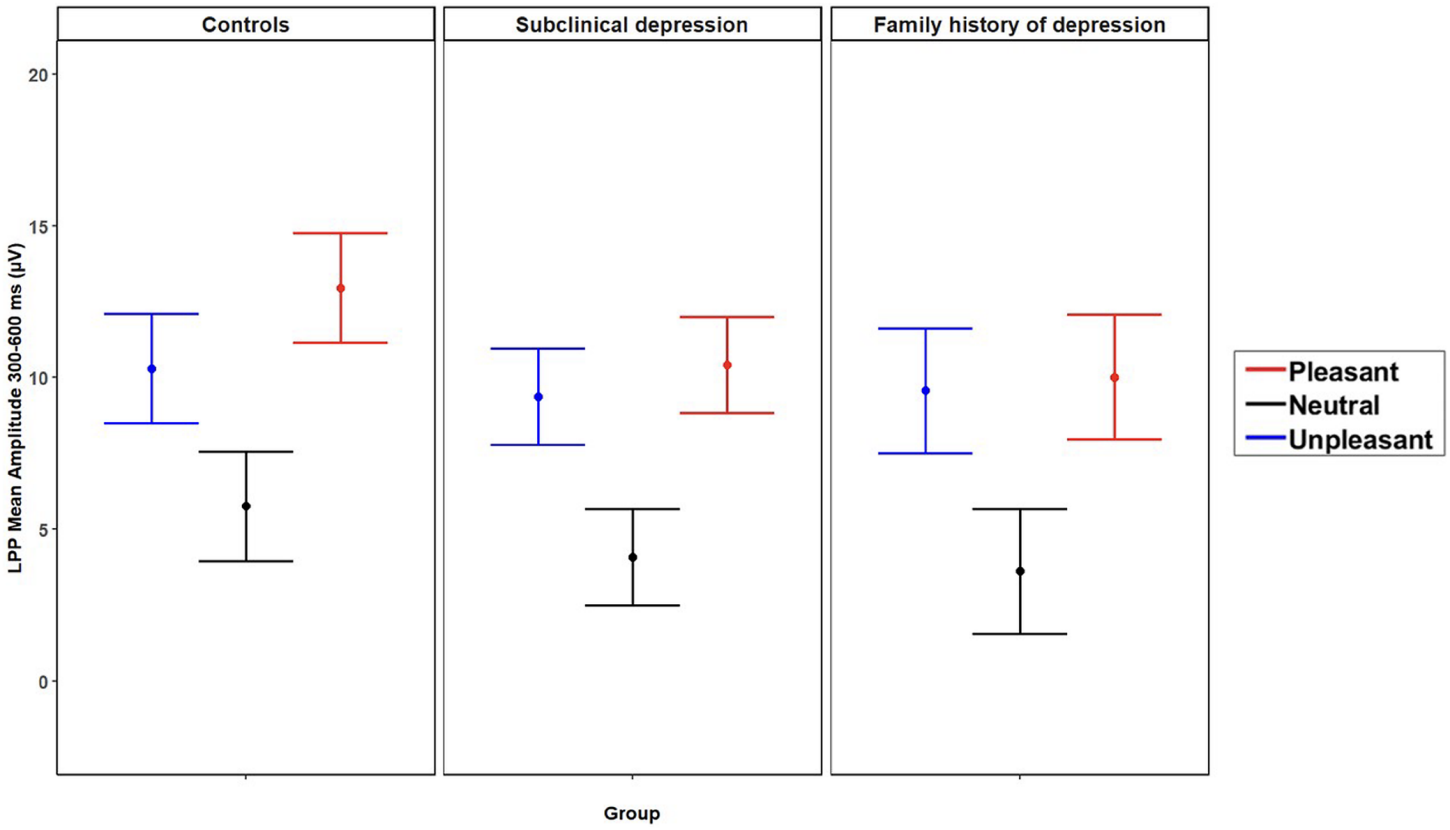
Mean amplitude of the LPP (300–600 ms) as a function of category (pleasant, neutral, unpleasant) during image (S2) presentation in the S1–S2 task, across the three groups: Control group (left), subclinical depression group (center), and familial risk group (right). Error bars represent the standard deviation around the mean.

In the first time window (300–600 ms), a significant effect was observed for the Group × Category interaction (see Figure 6). Post hoc within‐subjects comparisons of the significant Group × Category interaction indicated that, across all three groups, LPP amplitudes were significantly larger for pleasant and unpleasant images compared to neutral images (all *p*s < 0.001). Moreover, in the control and subclinical group, pleasant images showed a larger LPP than unpleasant ones (*p* < 0.02), while in the familial group this effect was not significant (*p* = 0.64). Post hoc between‐subjects comparisons revealed significantly smaller LPP amplitude in the two vulnerability groups compared to controls in the pleasant condition (all *p*s < 0.04). No other significant differences in LPP amplitude were observed.

In the second time window (600–1000 ms), a significant interaction effect was observed for the Group × Category interaction. Post hoc within‐subjects comparisons of the significant Group × Category interaction indicated that, across all three groups, LPP amplitudes were significantly larger for pleasant and unpleasant images compared to neutral images (all *p*s < 0.001). Moreover, in the subclinical and familial group, unpleasant images showed a larger LPP than pleasant ones (*p* < 0.02), while in the control group this effect was not significant (*p* = 0.45). Post hoc between‐subjects comparisons did not reveal any differences between the three groups (Figure S3).

In the last time window (1000–2000 ms), no significant Group × Category interaction emerged.

Additional analyses were conducted within the subclinical sample by stratifying participants based on the presence versus absence of a family history of depression. This approach was adopted to ensure that the observed group effect was not merely attributable to additive familial risk. We tested whether the two subgroups differed in ERP amplitudes across the three stimulus categories. As detailed in the Supporting Information, these analyses revealed no significant differences between participants with and without a family history of depression (Table S4 and Figures S4 and S5).

## 4 Discussion

The present study investigated distinct phases of emotional processing as potential psychophysiological correlates of vulnerability to depression, comparing two groups of young adults at elevated risk (individuals with a familial history of depression and those with subclinical depressive symptoms) to a control group without known risk. By leveraging the temporal precision of ERPs within an emotional S1–S2 paradigm, it was examined whether the two vulnerable groups exhibited changes in the anticipation (Cue‐P300, SPN) and elaboration (LPP) of emotional stimuli relative to low‐risk controls. Overall, results revealed that both vulnerability groups showed attenuated Cue‐P300 amplitudes during the anticipatory phase across emotional conditions, indicating reduced preparatory allocation of attentional resources to emotional stimuli. During stimulus processing, results indicated that group differences were most evident in the early LPP window (300–600 ms), where both at‐risk groups showed reduced LPP amplitudes to pleasant stimuli compared to controls. In later time windows, emotional modulation remained evident within groups, but between‐group differences were no longer significant.

As hypothesized, both vulnerability groups exhibited attenuated Cue‐P300 amplitudes relative to controls for pleasant and unpleasant trials. These findings suggest that individuals at risk for depression allocate fewer attentional resources to upcoming emotionally salient events than controls, reflecting diminished anticipation. This result is consistent with prior studies examining Cue‐P300 activity in monetary reward paradigms, which have shown that individuals with higher depressive symptoms exhibit reduced neural responses to reward‐predicting cues (Novak et al. 2016; Thompson et al. 2023). Such findings have been interpreted as evidence of diminished attentional engagement with motivationally significant stimuli. Importantly, the present study extends this literature, suggesting that Cue‐P300 reductions were evident not only for pleasant but also for unpleasant cues and, thus, that anticipatory deficits in depression vulnerability are not confined to appetitive contexts. Rather, they may reflect a more general attenuation in the preparatory mobilization of attention toward emotionally salient events, regardless of valence. At the same time, the within‐group pattern suggests that vulnerability to depression may also be associated with differences in how emotional cues are prioritized according to valence. In controls, both pleasant and unpleasant cues elicited larger Cue‐P300 amplitudes than neutral cues, and pleasant cues elicited larger amplitudes than unpleasant cues, suggesting both emotional salience detection and preferential engagement with pleasant cues. In the subclinical depression group, pleasant cues remained differentiated from both neutral and unpleasant cues, whereas unpleasant cues were only marginally differentiated from neutral cues. In the familial risk group, both pleasant and unpleasant cues were differentiated from neutral cues, but not from each other. Thus, the vulnerability groups did not show a complete absence of emotional cue discrimination. Instead, they showed a different organization of anticipatory attention, with weaker aversive‐cue differentiation in the subclinical group and reduced pleasant‐versus‐unpleasant differentiation in the familial‐risk group. This broader anticipatory blunting may represent an early change within both Positive and Negative Valence Systems, potentially limiting adaptive anticipation to respond to significant environmental challenges in individuals at elevated risk for developing depression.

Consistent with previous studies, at the level of stimulus processing, the early LPP window showed significantly reduced amplitudes to pleasant stimuli in both vulnerability groups compared to controls, whereas no group differences emerged for unpleasant or neutral stimuli. This valence‐specific blunting is in line with a robust body of literature documenting reduced LPP amplitudes to pleasant stimuli in individuals with clinical and subclinical depression (e.g., Angeleri et al. 2026; Dell'Acqua et al. 2026; Klawohn et al. 2021; Moretta et al. 2021; Moretta and Messerotti Benvenuti 2023) and with depression vulnerability (Mologni, Dell'Acqua, Mejza, et al. 2026), and supports the notion of diminished engagement with appetitive content. Importantly, the present findings indicate that reduced motivated attention toward pleasant stimuli is not only characteristic of individuals with current depressive symptoms, but is also observable in those with a familial risk for depression who do not report current symptomatology. Moreover, the present findings suggest that emotional processing in depression vulnerability may follow a different temporal profile across successive stages of stimulus elaboration. In the early 300–600 ms window, the main group difference concerned reduced processing of pleasant stimuli in both vulnerability groups. In the later 600–1000 ms window, however, between‐group differences were no longer significant, although the two vulnerability groups showed larger LPP amplitudes to unpleasant than pleasant images, a pattern not observed in controls. This suggests that vulnerability to depression may be characterized not only by reduced early engagement with pleasant content, but also by a later shift in emotional differentiation, with relatively greater elaboration of unpleasant compared to pleasant stimuli. Thus, the vulnerability groups did not simply show globally blunted emotional processing, but a different temporal organization of emotional attention, characterized by relatively weaker early prioritization of pleasant information and greater later differentiation in favor of unpleasant content.

Of note, no significant differences emerged between the two vulnerability groups in either the anticipatory (Cue‐P300) or stimulus‐processing (LPP) phases. Despite differing in their clinical profile (i.e., one group characterized by elevated subclinical depressive symptoms and the other by familial risk in the absence of current symptomatology), both exhibited a highly similar pattern of neurophysiological responses. This finding strengthens the interpretation that the observed psychophysiological changes do not merely reflect the state effects of current symptoms, but may instead represent trait‐like markers of vulnerability. Hence, the absence of group differences lends further support to the conceptualization of blunted anticipatory engagement and reduced motivated attention to pleasant stimuli as potential psychophysiological indicators of depression risk.

Within the RDoC framework, this pattern is consistent with the hypothesis of hypoactivation of the Positive Valence System as a central mechanism underlying vulnerability to depression. At the same time, the nonsignificant group differences in LPP amplitudes to unpleasant stimuli suggests that the ECI (Rottenberg et al. 2005) hypothesis may be only partially expressed at the vulnerability stage. While clinical depression has often been associated with more generalized attenuation of emotional responses across valence (e.g., Weinberg et al. 2016), our findings point to a more circumscribed alteration primarily affecting pleasant stimuli. Importantly, the lack of blunted responses to unpleasant stimuli in our at‐risk groups should not be interpreted as evidence that Negative Valence System functioning is uniformly preserved in vulnerability. Rather, it is plausible that the expression of negative valence alterations depends on moderating factors that were not directly examined in the present study. For instance, exposure to early adverse experiences has been shown to shape neural responses to emotional stimuli and may amplify the LPP to unpleasant stimuli, particularly in individuals with familial risk (e.g., Angeleri et al. 2026). Thus, variability in stress exposure or other developmental variables may account for inconsistencies across studies.

Regarding subjective measures, no significant group differences emerged in self‐reported valence or arousal ratings. This pattern is consistent with prior research in subclinical depression (Benning and Ait Oumeziane 2017; Moretta et al. 2021; Sloan and Sandt 2010), where blunted LPP to affective stimuli was observed in the absence of differences in subjective emotional ratings. Taken together, these findings suggest that the observed group differences in Cue‐P300 and LPP amplitudes cannot be attributed to variations in subjective perceptions of stimulus valence or arousal. Rather, they point to a dissociation between explicit emotional appraisal and underlying neural processing. Importantly, this pattern supports the notion that ERPs may represent more sensitive markers of vulnerability to depression than self‐report measures. Unlike subjective ratings, which capture consciously accessible aspects of emotional experience, electrophysiological indices provide access to rapid and potentially automatic stages of affective processing. As such, they may detect subtle alterations in attentional and motivational mechanisms that are not yet expressed at the level of conscious report, but that nonetheless reflect atypical affective processing in individuals at elevated risk for developing depression.

The present findings carry important clinical implications for early identification and prevention of depression. First, the combination of reduced anticipatory attention to all emotional stimuli (Cue‐P300) and diminished initial motivated attention to pleasant stimuli (LPP) highlights potential intervention targets. Preventive strategies could be designed to specifically enhance anticipatory motivation and engagement with positive experiences. Interventions such as behavioral activation, positive affect training, and reward‐based cognitive bias modification may directly address these motivational deficits (Craske et al. 2016, 2023). Also, ERPs could serve not only as risk indicators but also as objective outcome measures to evaluate changes in affective processing following an intervention. Finally, within stepped‐care or university‐based screening programs, incorporating brief EEG paradigms could contribute to a multi‐method assessment framework that integrates clinical interviews, self‐report, and psychophysiological indices. In the present study, group differences emerged in Cue‐P300 and early LPP amplitudes despite the absence of group differences in self‐reported valence and arousal ratings, suggesting that these measures may be sensitive to subtle differences in emotional processing. Thus, ERPs may be particularly useful for identifying mechanisms of risk and for tracking changes in affective processing in prevention or intervention settings. Future longitudinal work will be necessary to determine whether these ERP indices provide incremental predictive value over established clinical indicators, such as current symptoms and family history. Although further validation and longitudinal prediction are needed, the present results suggest that ERPs may help bridge basic psychophysiological research and clinical prevention by identifying early dysfunctions in motivational systems before the potential development of depression.

The present findings should be considered in light of several methodological limitations. First, the sample primarily consisted of university students and was predominantly female. Although sex distribution did not differ across groups, the overall overrepresentation of females may limit the generalizability of the findings, particularly to male populations or to more diverse community samples. Replication in more heterogeneous and gender‐balanced samples will be important to strengthen our findings. Second, the cross‐sectional design precludes conclusions regarding the predictive value of the observed ERP changes. Only longitudinal investigations can determine whether the reduced Cue‐P300 and LPP amplitudes identified here represent stable vulnerability markers that prospectively predict the onset or worsening of depressive symptoms, particularly in individuals with familial risk. Finally, the SPN component was not reliably observed, which limited the ability to fully characterize anticipatory processes. A similar absence of a SPN has been reported in a recent study employing the same S1–S2 paradigm (Mologni, Dell'Acqua, and Messerotti Benvenuti 2026), suggesting that task‐related features may constrain the elicitation of sustained expectancy‐related activity in this context. Future studies should consider refining task parameters, such as strengthening cue–stimulus contingencies, to enhance expectancy‐related neural activity and allow for a more comprehensive assessment of anticipatory mechanisms in depression vulnerability.

A further limitation concerns the partial overlap between familial vulnerability and current subclinical depressive symptoms. Although the family history group was defined by familial risk in the absence of current symptoms, a subset of participants in the subclinical depression group also reported a family history of depression. This overlap reflects the clinical reality that vulnerability factors often co‐occur, but it limits the extent to which familial risk and current symptom‐related vulnerability can be fully disentangled. Yet, sensitivity analyses included in the Supporting Information suggested that the ERP profile of the subclinical group was not exclusively driven by participants with co‐occurring family history. Finally, past depressive episodes were not an exclusion criterion and several participants in every group had experienced a depressive episode in the past, especially those with a family history for the disorder. Given the small and uneven number of participants with past depression across groups, subgroup analyses comparing remitted and never‐depressed participants were not conducted. Future studies with larger samples specifically designed to distinguish never‐depressed high‐risk individuals from individuals with remitted depression will be needed to clarify whether these ERP changes represent risk markers, residual effects of prior depression, or both.

Overall, the findings of the present study indicate that both familial risk for depression and subclinical depressive symptoms are characterized by attenuated anticipatory engagement with emotionally salient cues and reduced motivated attention toward pleasant stimuli in the initial stages of motivated attention. Specifically, at‐risk individuals showed diminished preparatory allocation of attentional resources during anticipation, together with blunted neural responses to pleasant content during stimulus processing. These patterns suggest that alterations in affective anticipation and positive emotional engagement may represent early psychophysiological features of vulnerability to depression. Such alterations were detectable even in the absence of clinically significant symptoms and despite intact subjective emotional ratings, underscoring their potential value as subtle markers of latent risk. Importantly, the lack of significant differences between the subclinical and familial risk groups is theoretically meaningful. Despite differences in current symptom levels, both groups displayed similar anticipation and elaboration patterns, suggesting that the observed ERP changes may index latent vulnerability rather than manifest depressive severity.

## Author Contributions

**Aurelia Lo Presti:** conceptualization, writing – review and editing. **Rita Bianca Ardito:** conceptualization, funding acquisition, writing – review and editing, supervision. **Mauro Adenzato:** conceptualization, funding acquisition, writing – review and editing, supervision. **Carola Dell'Acqua:** conceptualization, investigation, writing – original draft, methodology, visualization, writing – review and editing, software, formal analysis, data curation. **Valentina Mologni:** investigation, methodology, writing – review and editing. **Benedetto Farina:** conceptualization, funding acquisition, writing – review and editing, project administration, supervision, resources. **Giuseppe Alessio Carbone:** writing – review and editing, conceptualization. **Letizia Soliman:** investigation, methodology, writing – review and editing. **Claudio Imperatori:** conceptualization, funding acquisition, writing – review and editing, project administration, supervision, resources. **Simone Messerotti Benvenuti:** conceptualization, funding acquisition, writing – review and editing, methodology, project administration, data curation, supervision, resources.

## Funding

We acknowledge financial support from the National Recovery and Resilience Plan (NRRP), funded by the European Union—NextGenerationEU, for the project “Cognitive, affective, and neural mechanisms of depression vulnerability: searching for endophenotypes and risk factors” (Project No. 20228P4H2K), adopted by the Italian Ministry of University and Research (MUR). We also acknowledge financial support to Dr. Carola Dell'Acqua from the MUR under the Young Researchers 2024—Seal of Excellence 2024 program (Project title: “Uncovering dePREssion vulnerability: an investigation of social reWARD sensitivity in at‐risk adolescents (PREWARD)”), within the NRRP funded by the European Union—NextGenerationEU.

## Conflicts of Interest

The authors declare no conflicts of interest.

## Supporting information


**Figure S1:** Scalp topographies of LPP mean amplitudes in the 600–1000 ms time window following image, S2, onset during the S1–S2 task. Topographic maps are displayed separately for each group, controls, subclinical depression, and family history of depression, and for each image category, unpleasant, neutral, and pleasant. Warmer colors indicate larger positive amplitudes in μV.
**Figure S2:** Scalp topographies of LPP mean amplitudes in the 1000–2000 ms time window following image, S2, onset during the S1–S2 task. Topographic maps are displayed separately for each group, controls, subclinical depression, and family history of depression, and for each image category, unpleasant, neutral, and pleasant. Warmer colors indicate larger positive amplitudes in μV.
**Figure S3:** Mean amplitude of the LPP (600–1000 ms) as a function of Category (pleasant, neutral, unpleasant) during image (S2) presentation in the S1–S2 task, across the three groups: control group (left), subclinical depression group (center), and familial risk group (right). Error bars represent the standard deviation around the mean.
**Figure S4:** Grand average ERP waveforms during the S1–S2 task presented for the average of parietal electrodes (P3, PZ, P4). Cue (S1) onset was at 0 s (dotted line) in the sample with subclinical depression with (*n* = 18) and without (*n* = 21) family history of depression.
**Figure S5:** Grand average ERP waveforms during the S1–S2 task presented for the average of parietal electrodes (P3, PZ, P4). Image (S2) onset was at 0 s (dotted line) in the sample with subclinical depression with (*n* = 18) and without (*n* = 21) family history of depression.
**Table S1:** Means and standard deviation of number (%) of rejected cue‐ and image‐locked epochs across the three groups.
**Table S2:** Internal consistency reliability indices (Cronbach's *α* and 95% confidence intervals, CI) for each ERP component and emotional category.
**Table S3:** Zero‐order correlations between differential scores of ERPs (pleasant—neutral, unpleasant—neutral) and depressive symptoms, age, and sex.
**Table S4:** ANOVA summary of the linear mixed‐effects models predicting ERPs (Cue‐P300 and LPP) using two groups (subclinical depression with familiarity, subclinical depression without familiarity).

## Data Availability

Data and analytical code are provided in the Open Science Framework (https://osf.io/sybdu/overview/) associated with this project.

## Endnotes

^1^
A subset of this sample (*n* = 42, 20 controls and 22 with subclinical depression) was previously reported in Dell'Acqua et al. (2025).

^2^
IAPS codes: 1302, 2396, 4611, 4645, 4647, 4651, 4652, 4660, 4664, 4670, 4680, 4683, 4690, 4695, 4810, 8030, 8031, 8034, 8080, 8161, 8180, 8185, 8186, 8200, 8280, 8370, 8400, 8490, 9425, 1050, 1114, 1120, 1275, 1300, 1930, 1932, 3500, 6200, 6210, 6230, 6242, 6243, 6244, 6250, 6260, 6312, 6313, 6370, 6510, 6540, 6550, 6560, 6825, 2102, 2383, 2393, 2493, 2495, 2575, 2595, 7000, 7002, 7004, 7009, 7010, 7020, 7035, 7036, 7041, 7050, 7056, 7059, 7130, 7175, 7224, 7233, 7242, 7491, 7500, 7547, 7560, 7595, 7700, 7950.
