# Supplementary material for: Affective Processing in Depression Risk: Insights From Event‐Related Potentials
Source: Psychophysiology. 2026 Jul 27;63(7):e70367. doi: 10.1111/psyp.70367 (PMC13408224; doi:10.1111/psyp.70367)
Supplement: Supplementary file 1 — Figure S1: Scalp topographies of LPP mean amplitudes in the 600–1000 ms time window following image, S2, onset during the S1–S2 task. Topographic maps are displayed separately for each group, controls, subclinical depression, and family history of depression, and for each image category, unpleasant, neutral, and pleasant. Warmer colors indicate larger positive amplitudes in μV. Figure S2: Scalp topographies of LPP mean amplitudes in the 1000–2000 ms time window following image, S2, onset during the S1–S2 task. Topographic maps are displayed separately for each group, controls, subclinical depression, and family history of depression, and for each image category, unpleasant, neutral, and pleasant. Warmer colors indicate larger positive amplitudes in μV. Figure S3: Mean amplitude of the LPP (600–1000 ms) as a function of Category (pleasant, neutral, unpleasant) during image (S2) presentation in the S1–S2 task, across the three groups: control group (left), subclinical depression group (center), and familial risk group (right). Error bars represent the standard deviation around the mean. Figure S4: Grand average ERP waveforms during the S1–S2 task presented for the average of parietal electrodes (P3, PZ, P4). Cue (S1) onset was at 0 s (dotted line) in the sample with subclinical depression with (n = 18) and without (n = 21) family history of depression. Figure S5: Grand average ERP waveforms during the S1–S2 task presented for the average of parietal electrodes (P3, PZ, P4). Image (S2) onset was at 0 s (dotted line) in the sample with subclinical depression with (n = 18) and without (n = 21) family history of depression. Table S1: Means and standard deviation of number (%) of rejected cue‐ and image‐locked epochs across the three groups. Table S2: Internal consistency reliability indices (Cronbach's α and 95% confidence intervals, CI) for each ERP component and emotional category. Table S3: Zero‐order correlations between differential scores of ERPs (pleasant—neutral, [file PSYP-63-e70367-s001.docx]

**Supplementary material**

**Affective processing in depression risk: Insights from ERPs**

**Table S1**. Means and standard deviation of number (%) of rejected cue- and image-locked epochs across the three groups.

|  | **Controls**  **(*n* = 30)** | | | **Subclinical depression**  **(*n* = 39)** | | | **Family history of depression**  **(*n* = 23)** | | |
| --- | --- | --- | --- | --- | --- | --- | --- | --- | --- |
|  | *Pleasant* | *Neutral* | *Unpleasant* | *Pleasant* | *Neutral* | *Unpleasant* | *Pleasant* | *Neutral* | *Unpleasant* |
| **Cue-locked** | 12.0 (12.3) | 11.2 (11.1) | 12.1  (13.6) | 11.0 (10.5) | 11.5 (9.41) | 11.3  (8.80) | 6.23 (6.89) | 6.09 (9.33) | 8.00  (11.7) |
| **Image-locked** | 3.67 (6.70) | 3.58 (4.47) | 3.13  (5.49) | 2.14 (3.75) | 3.33 (4.19) | 2.83  (4.49) | 2.74 (6.23) | 1.02 (2.43) | 2.22  (5.20) |

**Table S2.** Internal consistency reliability indices (Cronbach's α and 95% confidence intervals, CI) for each ERP component and emotional category.

|  |  | Cronbach's α | 95% CI |
| --- | --- | --- | --- |
| Cue-P300 | Pleasant | 0.69 | [0.60, 0.78] |
|  | Neutral | 0.53 | [0.39, 0.66] |
|  | Unpleasant | 0.62 | [0.51, 0.73] |
| LPP  (300 ms – 600 ms) | Pleasant | 0.85 | [0.81, 0.89] |
|  | Neutral | 0.83 | [0.78, 0.88] |
|  | Unpleasant | 0.87 | [0.83, 0.90] |
| LPP  (600 ms – 1000 ms) | Pleasant | 0.78 | [0.72, 0.84] |
|  | Neutral | 0.65 | [0.55, 0.75] |
|  | Unpleasant | 0.84 | [0.79, 0.88] |
| LPP  (1000 ms – 2000 ms) | Pleasant | 0.66 | [0.56, 0.76] |
|  | Neutral | 0.55 | [0.42, 0.68] |
|  | Unpleasant | 0.67 | [0.58, 0.77] |

**Table S3.** Zero-order correlations between differential scores of ERPs (pleasant – neutral, unpleasant – neutral) and depressive symptoms, age, and sex.

| **Predictor** | **ERP differential score** | **r** | ***p*** |
| --- | --- | --- | --- |
| BDI total | Cue-P300 NEG-NEU | -0.14 | .19 |
| BDI anhedonia | Cue-P300 NEG-NEU | -0.17 | .12 |
| Age | Cue-P300 NEG-NEU | -0.05 | .66 |
| Sex | Cue-P300 NEG-NEU | -0.05 | .66 |
| BDI total | Cue-P300 POS-NEU | -0.10 | .35 |
| BDI anhedonia | Cue-P300 POS-NEU | -0.11 | .32 |
| Age | Cue-P300 POS-NEU | -0.12 | .25 |
| Sex | Cue-P300 POS-NEU | -0.01 | .94 |
| BDI total | LPP 300-600 ms NEG-NEU | -0.02 | .84 |
| BDI anhedonia | LPP 300-600 ms NEG-NEU | 0.01 | .96 |
| Age | LPP 300-600 ms NEG-NEU | 0.04 | .73 |
| Sex | LPP 300-600 ms NEG-NEU | -0.25 | .02 |
| BDI total | LPP 300-600 ms POS-NEU | -0.16 | .13 |
| BDI anhedonia | LPP 300-600 ms POS-NEU | -0.16 | .12 |
| Age | LPP 300-600 ms POS-NEU | -0.11 | .31 |
| Sex | LPP 300-600 ms POS-NEU | -0.02 | .86 |
| BDI total | LPP 600-1000 ms NEG-NEU | -0.04 | .73 |
| BDI anhedonia | LPP 600-1000 ms NEG-NEU | 0.05 | .63 |
| Age | LPP 600-1000 ms NEG-NEU | 0.06 | .54 |
| Sex | LPP 600-1000 ms NEG-NEU | -0.14 | .20 |
| BDI total | LPP 600-1000 ms POS-NEU | -0.19 | .07 |
| BDI anhedonia | LPP 600-1000 ms POS-NEU | -0.15 | .16 |
| Age | LPP 600-1000 ms POS-NEU | 0.10 | .34 |
| Sex | LPP 600-1000 ms POS-NEU | -0.02 | .85 |
| BDI total | LPP 1000-2000 ms NEG-NEU | -0.04 | .69 |
| BDI anhedonia | LPP 1000-2000 ms NEG-NEU | -0.02 | .86 |
| Age | LPP 1000-2000 ms NEG-NEU | 0.03 | .78 |
| Sex | LPP 1000-2000 ms NEG-NEU | -0.14 | .17 |
| BDI total | LPP 1000-2000 ms POS-NEU | -0.17 | .11 |
| BDI anhedonia | LPP 1000-2000 ms POS-NEU | -0.18 | .08 |
| Age | LPP 1000-2000 ms POS-NEU | 0.10 | .32 |
| Sex | LPP 1000-2000 ms POS-NEU | 0.02 | .85 |

*Note. ERP differential scores were computed as emotional minus neutral amplitudes. Sex was coded as 0 = female and 1 = male. BDI = Beck Depression Inventory; LPP = late positive potential; NEG = negative; NEU = neutral; POS = positive.*


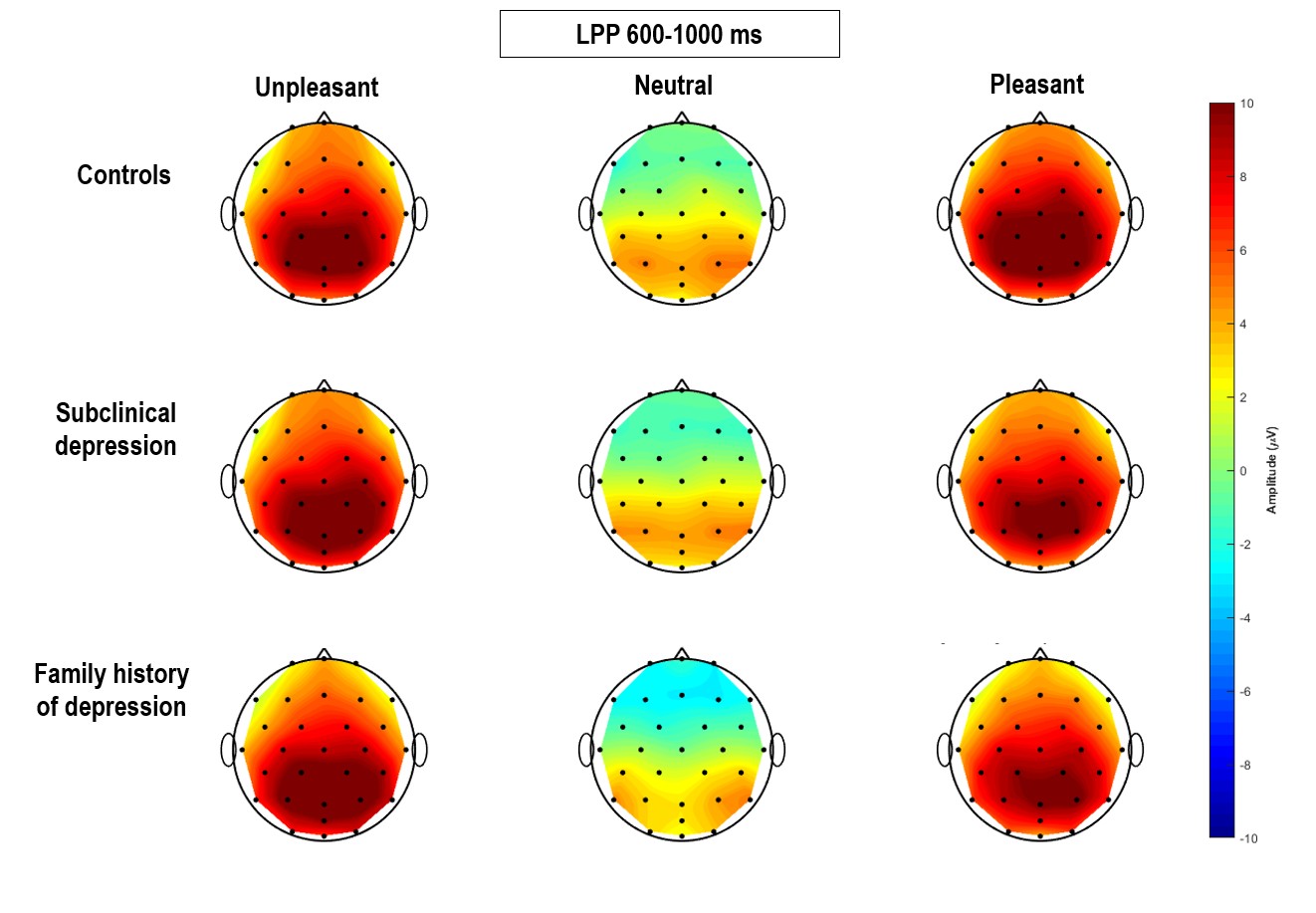


**Figure S1.** Scalp topographies of LPP mean amplitudes in the 600–1000 ms time window following image, S2, onset during the S1–S2 task. Topographic maps are displayed separately for each group, controls, subclinical depression, and family history of depression, and for each image category, unpleasant, neutral, and pleasant. Warmer colors indicate larger positive amplitudes in µV.


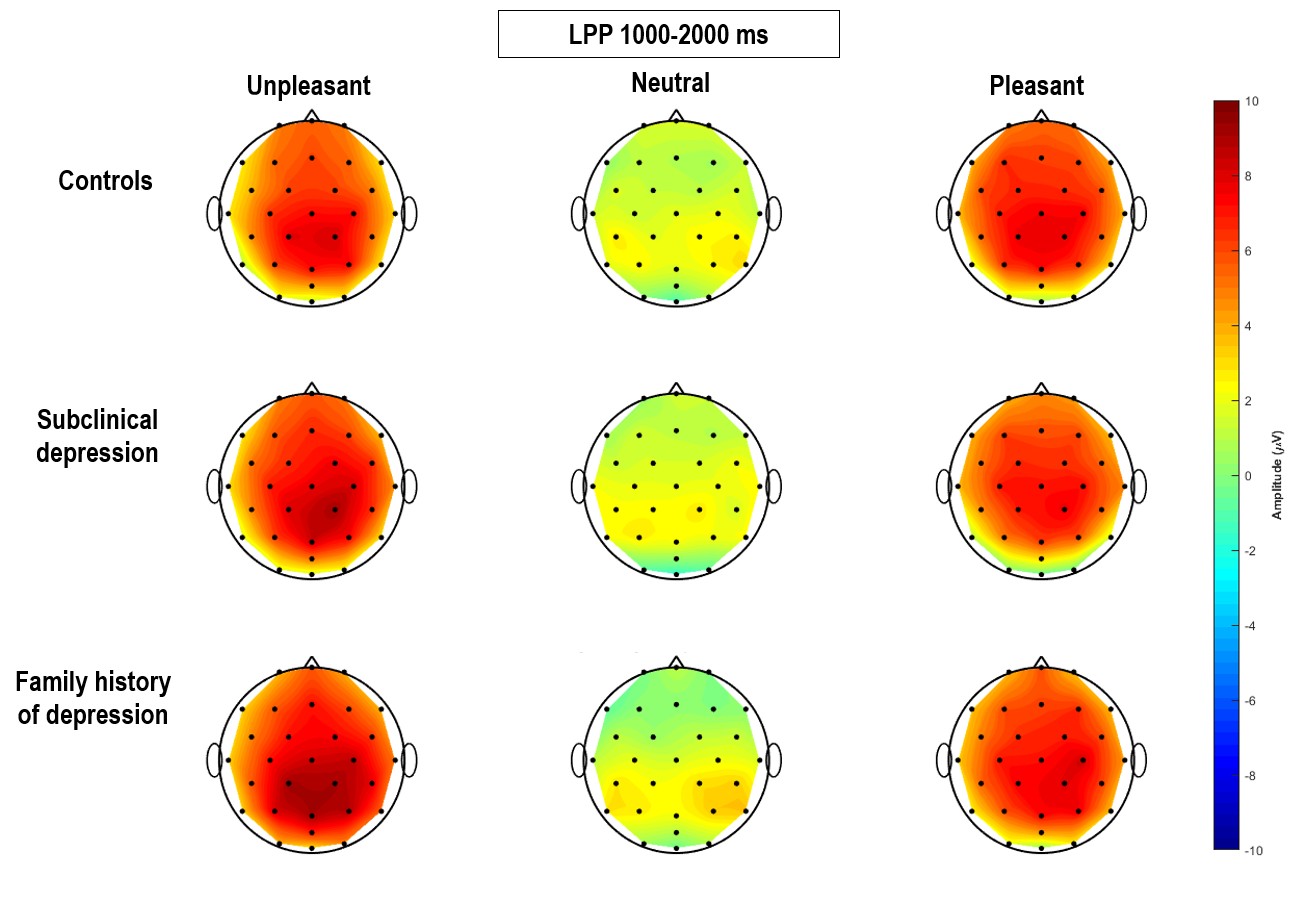


**Figure S2.** Scalp topographies of LPP mean amplitudes in the 1000–2000 ms time window following image, S2, onset during the S1–S2 task. Topographic maps are displayed separately for each group, controls, subclinical depression, and family history of depression, and for each image category, unpleasant, neutral, and pleasant. Warmer colors indicate larger positive amplitudes in µV.

**
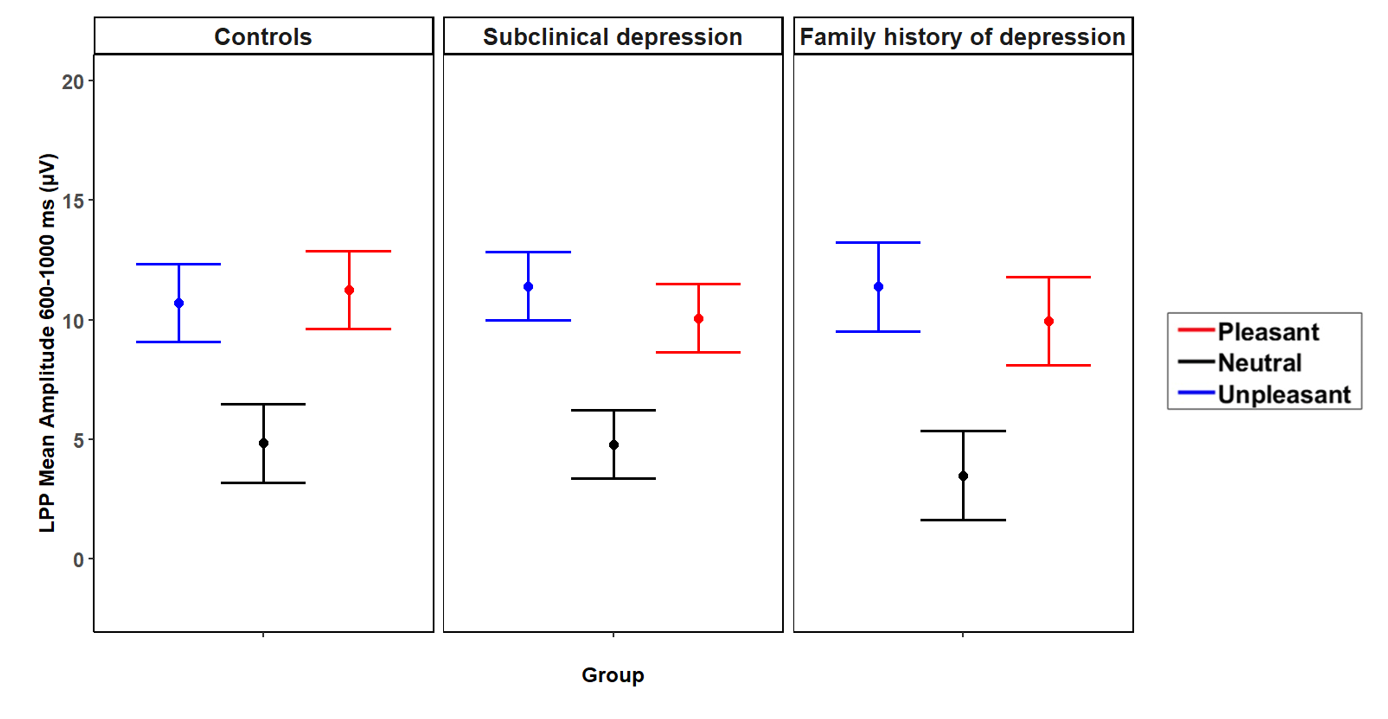
Figure S3.** Mean amplitude of the LPP (600-1000 ms) as a function of Category (pleasant, neutral, unpleasant) during image (S2) presentation in the S1–S2 task, across the three groups: control group (left), subclinical depression group (center), and familial risk group (right). *Note.* Error bars represent the standard deviation around the mean.

*Additional analyses were conducted within the subclinical sample by stratifying participants based on the presence versus absence of a family history of depression. This approach was adopted to ensure that the observed group effect was not merely attributable to additive familial risk. We tested whether the two subgroups differed in ERP amplitudes across the three stimulus categories. These analyses revealed no significant differences between participants with and without a family history of depression.*

**Emotional anticipation (Cue-P300) and elaboration (LPP) in the subclinical group with vs. without family history of depression.** Table S4 shows the results of the linear mixed-effects models examining Cue-P300 and LPP amplitudes as a function of Category, family history subgroup (subclinical depression with vs. without family history), and their interaction. Across all models, a significant main effect of Category emerged, indicating that ERP amplitudes varied as a function of stimulus category. However, neither the main effect of family history subgroup nor the Category × family history subgroup interaction was significant for the Cue-P300 or for any LPP time window. Post-hoc between-group comparisons confirmed that subclinical participants with versus without family history did not differ significantly in ERP amplitude for any stimulus category, negative, neutral, or positive, across all components and time windows. Overall, these findings suggest that, within the subclinical depression group, the ERP profile was not significantly different between participants with and without a family history of depression. Thus, the pattern observed in the subclinical group does not appear to be exclusively driven by participants with co-occurring familial risk.**
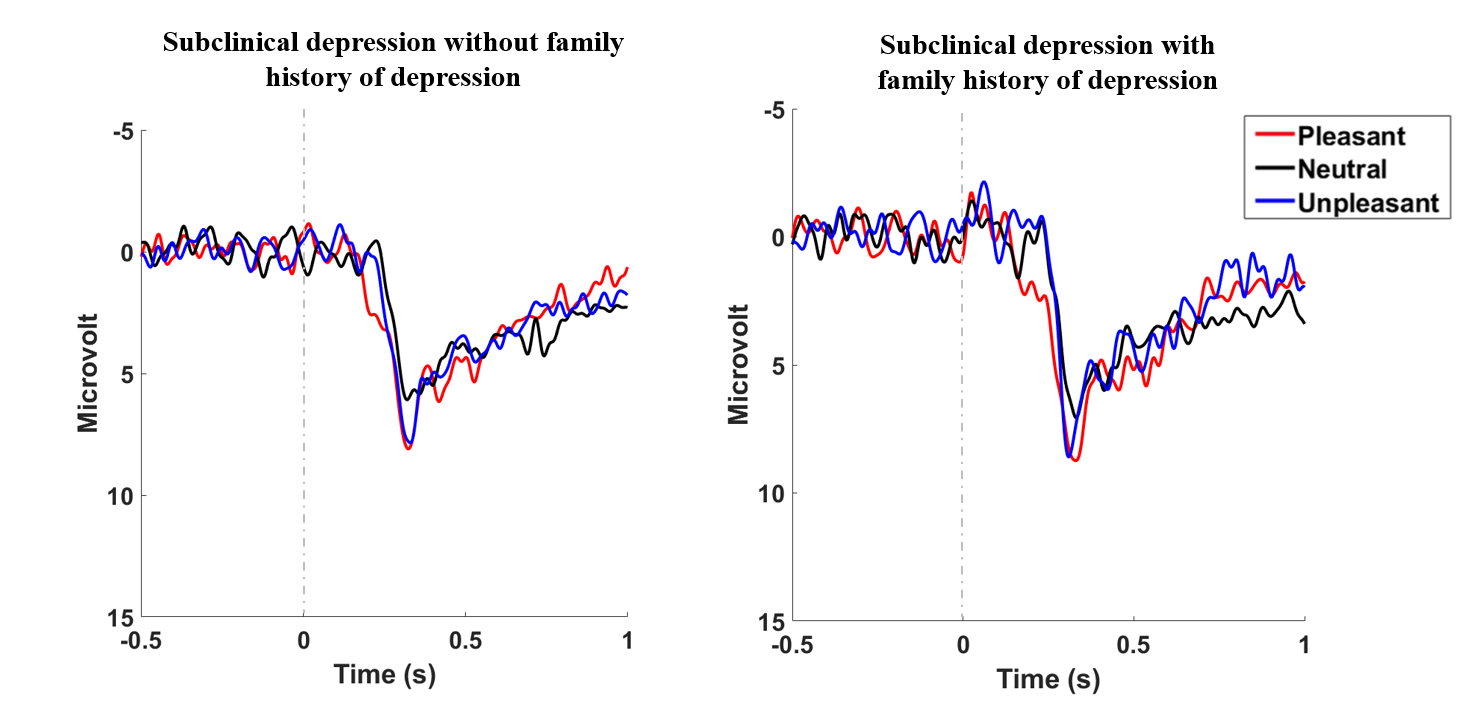
**

**Figure S4.** Grand average ERP waveforms during the S1-S2 task presented for the average of parietal electrodes (P3, PZ, P4). Cue (S1) onset was at 0 s (dotted line) in the sample with subclinical depression with (*n* = 18) and without (*n* = 21) family history of depression.

**
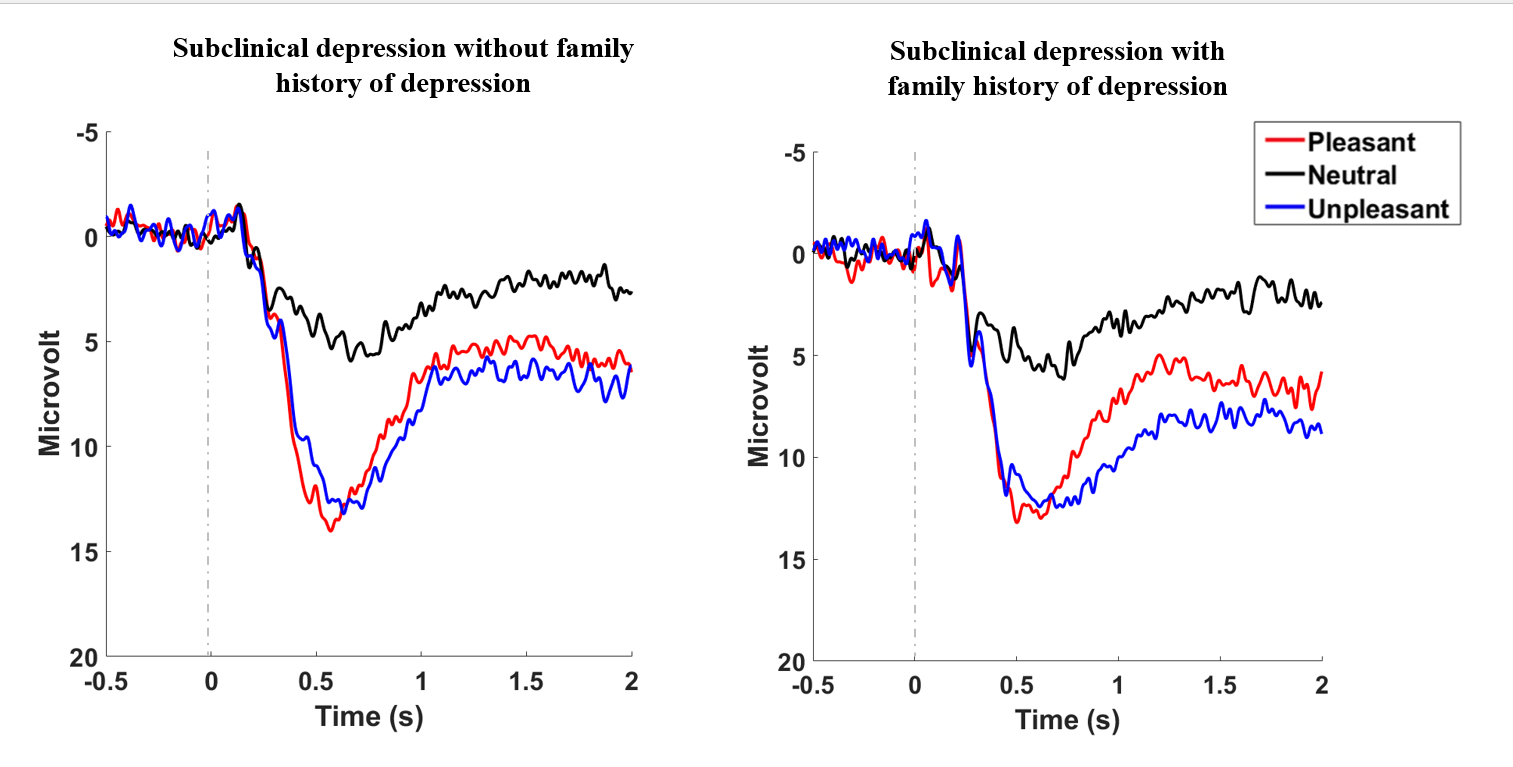
**

**Figure S5.** Grand average ERP waveforms during the S1-S2 task presented for the average of parietal electrodes (P3, PZ, P4). Image (S2) onset was at 0 s (dotted line) in the sample with subclinical depression with (*n* = 18) and without (*n* = 21) family history of depression.

**Table S4.** ANOVA summary of the linear mixed-effects models predicting ERPs (Cue-P300

and LPP) using two groups (subclinical depression with familiarity, subclinical depression without familiarity).

| **Cue-P300 model** | | | |
| --- | --- | --- | --- |
|  | ***df*** | ***F*** | ***p-value*** |
| **Category** | **2** | **23.03** | **< .001** |
| Group | 1 | 0.08 | .78 |
| Group × Category | 2 | 1.81 | .16 |
| **LPP model (300-600 ms)** | | | |
|  | ***df*** | ***F*** | ***p-value*** |
| **Category** | **2** | **166.28** | **< .001** |
| Group | 1 | 0.03 | .87 |
| Group × Category | 2 | 0.73 | .48 |
| **LPP model (600-1000 ms)** | | | |
|  | ***df*** | ***F*** | ***p-value*** |
| **Category** | **2** | **138.14** | **< .001** |
| Group | 1 | 0.03 | .86 |
| Group × Category | 2 | 0.64 | .53 |
| **LPP model (1000-2000 ms)** | | | |
|  | ***df*** | ***F*** | ***p-value*** |
| **Category** | **2** | **67.99** | **< .001** |
| Group | 1 | 0.41 | .52 |
| Group × Category | 2 | 2.15 | .12 |

*Note.* Significant effects are shown in bold. Df = degrees of freedom.
